# Venereological practice and the *Patronato de Protección a la Mujer* (patronage for the protection of women) in Catalonia during the Franco Dictatorship (1939–1975)

**DOI:** 10.3389/fpubh.2026.1794738

**Published:** 2026-06-17

**Authors:** Florian Grafl, Florian Steger

**Affiliations:** Institute of the History, Philosophy and Ethics of Medicine, Ulm University, Ulm, Germany

**Keywords:** adolescents, epidemiology, history, public health, sexually transmitted diseases, Spain, venereology

## Abstract

**Background:**

Since early modern times, people suffering from venereal diseases have been subject to social stigmatization. In the German Democratic Republic and in other socialist dictatorships, young women who did not conform to the regime’s ideology had to undergo medically unnecessary venereological treatments. At the beginning of the Franco Dictatorship in Spain (1939–1975), the *Patronato de Protección a la mujer* (Patronage for the Protection of Women) was created, aiming to discipline female adolescents. The state Catholic organization had its own medical service. This paper examines the institutions of the Patronato in Catalonia in order to determine if its venereological practice was influenced by Francoist ideology.

**Methods:**

We analyzed contemporary publications of the Patronato’s national board from the National Catalan Archive in Sant Cugat de Vallès as well as unpublished documents of the Patronato’s provincial boards in Catalonia from the Archive of the Spanish Government Representation in Barcelona and the Historical Archive of Lleida. To examine these sources, we implemented the historical-critical method.

**Results:**

At the headquarters of the provincial board of the Patronato in Barcelona, there was a treatment room where venereological examinations were carried out against the will of the patients. The Patronato cooperated with the *Hospital de la Magdalena*, a closed venereology ward to which women with venereal diseases were forcibly admitted for inpatient treatment until this institution was closed in 1959. Since 1963, young women who had been handed over to the Patronato were isolated in a so-called *Centro de Observación y Clasificación* (Observation and Classification Centre), where they had to undergo extensive medical examinations to determine their further treatment.

**Conclusion:**

In all three of the institutions examined in this study, examinations were carried out on the basis of questionable medical indications. The patients were young women who had been referred to the Patronato because they had violated the social norms of the Franco regime. This suggests that the examinations were less a part of medical care and more a part of social control mechanisms.

## 1 Introduction

**Figure 1:**
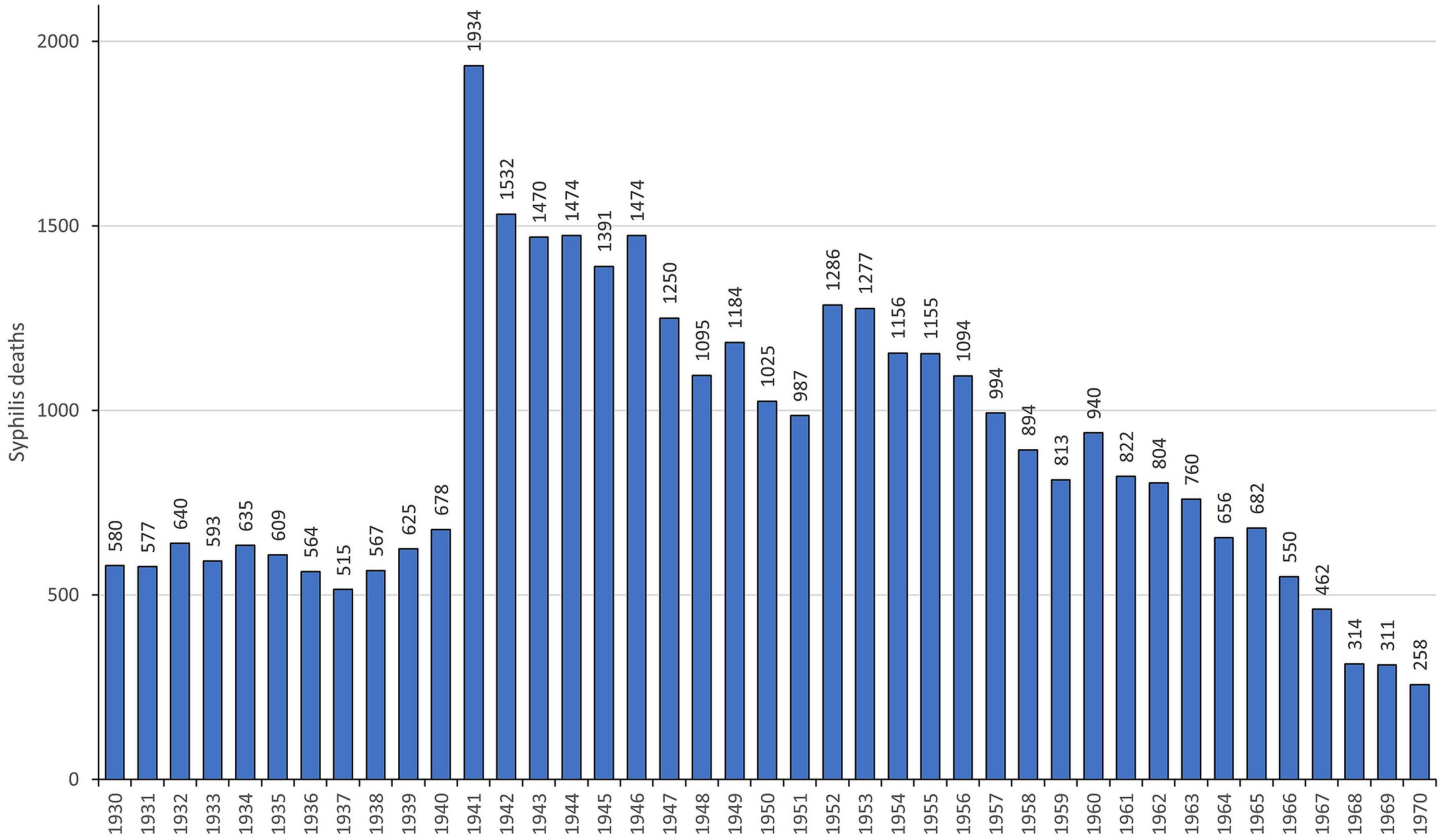
Mortality from syphilis in Spain between 1930 and 1970 (40).

The Spanish Civil War (1936–1939) had caused poor hygiene conditions and deficiencies in medical care for the population, which resulted in a serious deterioration of the health situation in Spain during the immediate post-war period. These conditions strongly favored the spread of infectious diseases and mortality rate increased significantly (1). This is particularly evident in the case of syphilis (Figure 1). The mortality rate was by far the highest shortly after the end of the Spanish Civil War and returned to its pre-war level only in the second half of the 1960s.

Consequently, since the beginning of the Franco Dictatorship (1939–1975), the fight against venereal diseases had been a priority in the regime’s health policy (2). In March 1941, the regulation of prostitution was reintroduced, which meant that registered prostitutes had to undergo regular venereological examinations (3). In medical circles, prostitutes who did no register and thus avoided the medical controls were primarily blamed for the spread of sexually transmitted diseases (4). This form of prostitution, known as *prostitución clandestina* (clandestine prostitution) was practiced by many underage sex workers, who engaged in prostitution for economic reasons but who, due to their age, could not officially work as prostitutes (5). To discourage them from their activity, a decree was enacted in November 1941 to put female adolescents suspected of engaging in clandestine prostitution under the guardianship of the Catholic organization *Patronato de Protección a la Mujer* (Patronage for the Protection of Women) (6). This article focuses on the issue of medical care, particularly with regard to the treatment of venereal diseases, provided by the venereologists at the service of the Patronato and the collaborating medical facilities. Although research on the Patronato and its institutions has grown considerably in recent years, this aspect has been studied very little so far (7, 8). This paper examines the medical service provided by the national board of the Patronato, as well as the venereological practice in the facilities of the provincial boards in Catalonia. The key question here is whether the examinations and treatments carried out there were medically justified or part of social control mechanisms.

## 2 Materials and methods

We analyzed contemporary legal texts, articles in medical journals and newspaper reports, publications of the Patronato’s national board in Madrid as well as unpublished documents of its provincial boards in Catalonia.

These sources mainly consist of directives sent by the national board of the Patronato to the provincial boards in all regions of Spain, of reports that the provincial boards had to send regularly to Madrid as well as the minutes of the commission meetings of the provincial boards. They can be located in the *Arxiu Nacional de Catalunya* (National Catalan Archive) in Sant Cugat de Vallès, the *Archivo de la Subdelegación del Gobierno* (Archive of the Spanish Government Representation) in Barcelona and the *Arxiu Històric* (Historical Archive) in Lleida.

To examine these records, we implemented the historical-critical method. The aim of this method is to understand and interpret historical documents as sources for reconstructing the past. This method places particular emphasis on the history of the sources to be analyzed and their integration into the historical context. The historical-critical method is carried out in three consecutive steps, which we applied to our materials in order to arrive at the findings of this paper: Firstly, we carefully selected the sources relevant to the research questions of this paper. Secondly, we critically examined what data could be reliably derived from the respective sources. Thirdly, we interpreted the sources to reconstruct the organization of the provincial boards of the Patronato in Catalonia as well as the venereological examinations and treatments carried out by doctors at the service of the Patronato and in medical facilities cooperating with the Patronato in Catalonia.

## 3 Results

### 3.1 The medical service of the Patronato’s national board in Madrid

The psychiatrist Dr. Francisco Javier de Echalecu y Canino (1897–1957) served as head of the medical service of the Patronato’s national board in Madrid. In 1949, he issued a directive to the provincial boards specifying the medical tasks they had to perform. Accordingly, an initial examination of the young women interned by the Patronato should determine whether they suffer from chronic, incurable or contagious diseases which could pose a danger to other inmates. A positive diagnosis of syphilis or gonorrhea had to be recorded in the medical history summary, along with the results of all the tests which were carried out, prescribed medication and doses administered (9).

**Figure 2:**
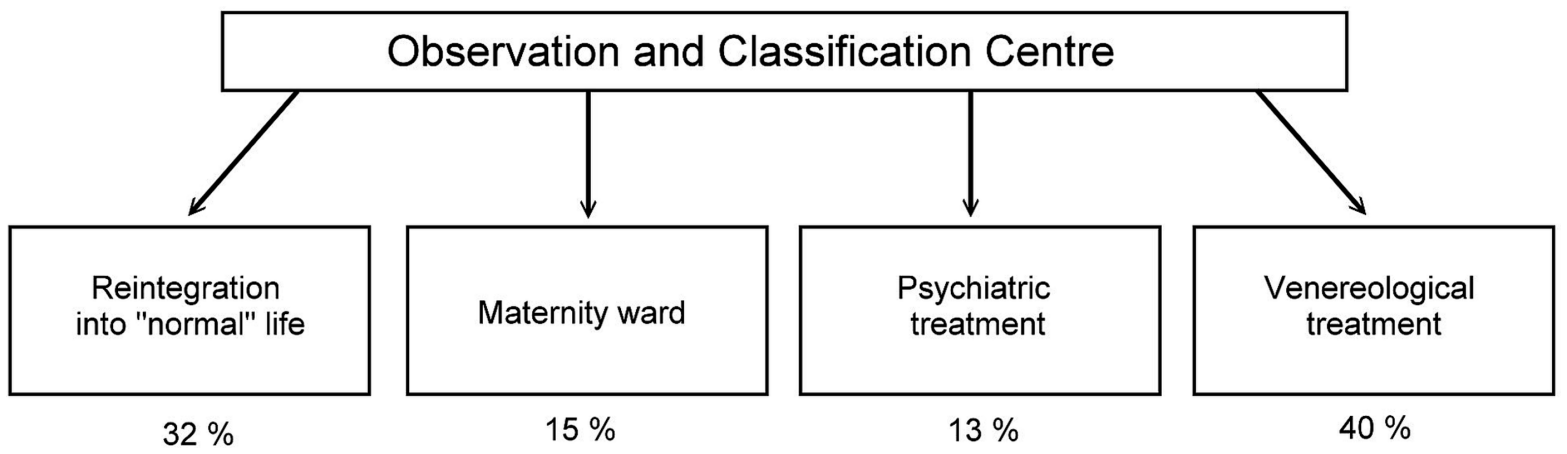
Chart showing the distribution of young women interned by the Patronato after examinations at the Observation and Classification Centre (41).

In the 1960s, the national board of the Patronato began to force the establishment of so-called *Centros de Observación y Clasificación* (Observation and Classification Centres) in metropolitan areas such as Madrid, Seville, Zaragoza and Barcelona. The medical staff of these centers consisted of a general practitioner, a psychologist, a psychiatrist, a venereologist, and a nurse. Young women who were handed over to the Patronato by the police or the authorities were initially isolated there for several weeks. Extensive psychological and medical examinations determined whether they were pregnant, had any sexually transmitted diseases or had to be categorized as “homosexual,” “abnormal,” or “rebellious,” which, according to the Francoist ideology propagated by the Patronato, were considered as mental disorders. Based on the results of these investigations, it was determined to which institutions the young women should then be referred to. According to a publication by the Patronato’s national board from 1965, after their initial examinations in the Observation and Classification Centres the majority of the young women received venereological treatment (Figure 2). It is remarkable in that, as explained in the introduction, the mortality rate from syphilis had already fallen significantly by the mid-1960s compared with the early years of the Franco dictatorship. This can be explained by the fact that the decision regarding the young women in question was not based on medical grounds. Instead, all young women who were referred to the Patronato and suspected of engaging in prostitution were subjected to a venereological examination.

### 3.2 Venereological examinations at the headquarters of the Patronato’s provincial board in Barcelona

**Figure 3:**
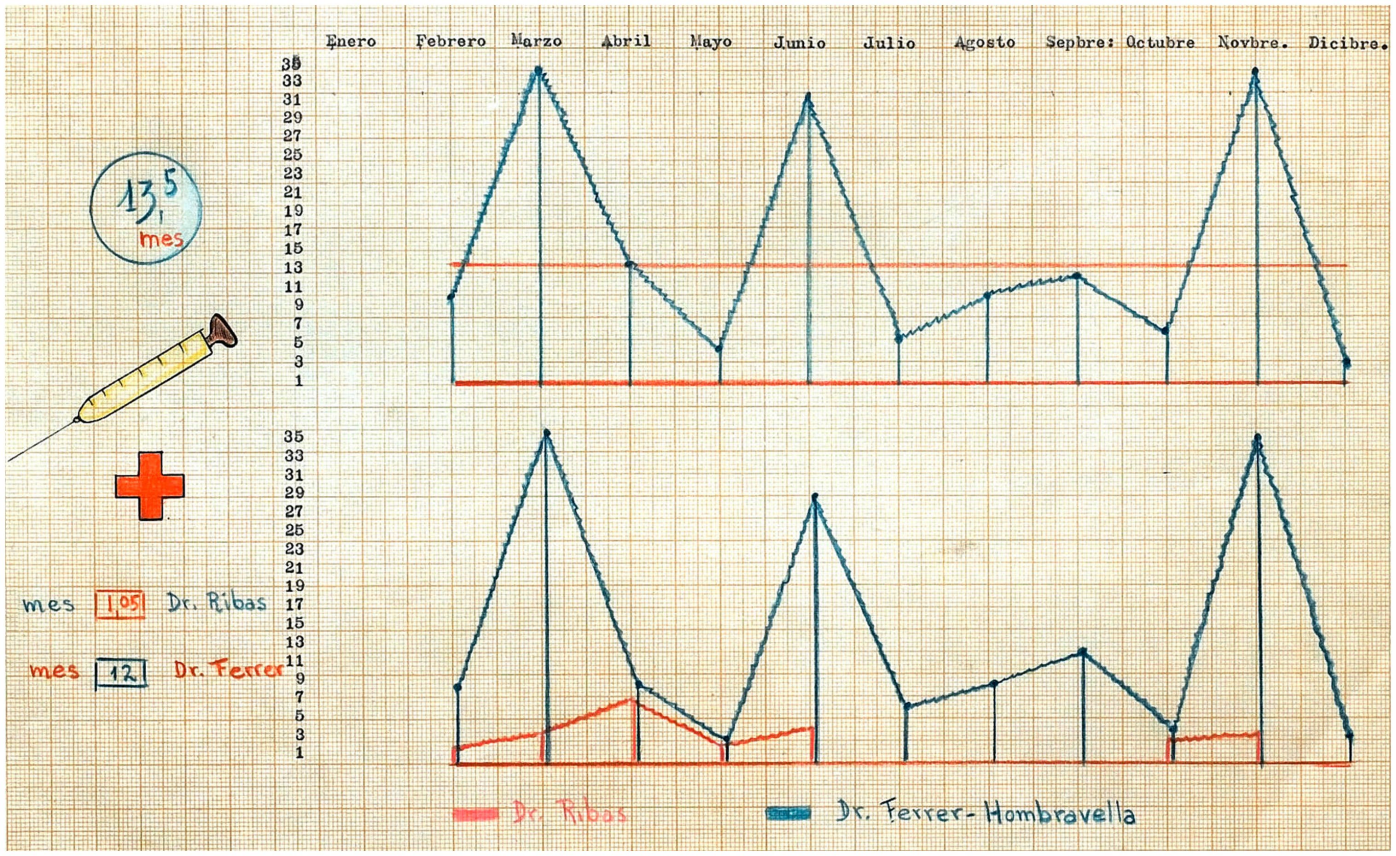
Statistics on monthly medical services at the headquarters of the Provincial Board of the Patronato in Barcelona for the year 1957 (42).

The provincial board of the Patronto in Barcelona was founded in April 1943 (10). Its medical staff consisted of a general practitioner, who also had to be specialized in gynecology, a dermatologist and a psychiatrist (11). In December 1952, the provincial board of the Patronato in Barcelona moved to a new headquarter which also had a room designated for “medical services” (12). There are only two documents available that provide information about which medical examinations and treatments were conducted there. They show that in 1957, the services of “Dr Ribas” were used on average once and those of “Dr Ferrer-Hombravella” on average 12 times per month (Figure 3).

Such statistics are also available for the following year. However, as these are incomplete, they can only be seen as evidence that both doctors continued their medical activities at the headquarters of the Patronato in 1958 (13). Although, according to these statistics, significantly more young women received psychiatric treatment, venereological examinations were also carried out regularly, on average once a month. Dr. José Ferrer-Hombravella worked as a psychiatrist for the provincial board of the Patronato in Barcelona until 1962 (14). Dr. Juan Ribas Magri originated from a prestigious Catalan family of doctors. Within the provincial board of the Patronato, he was designated as obstetrician and „specialist in sexual manners “(15). In a police report from 1953, he was accused of examining young women against their will and the consent of their mothers to determine whether they were still virgins. These examinations were used in conjunction with other questionable interrogation methods to make two young women confess that they had behaved inappropriately according to the traditional, Catholic image of gender propagated by the Franco regime (50).

Ribas Magri’s services for the Patronato are documented until at least the beginning of 1967 (16). During that period of time, he was involved in the provincial board of the Patronato in Barcelona far beyond his role as a venereologist. Since January 1944, he regularly attended its commission meetings (17). Later he even became a permanent member of its steering committee for several years (18).

### 3.3 The Observation and Classification Centre in Barcelona

In 1963, an Observation and Classification Centre was opened in Barcelona under the name *Centro de Orientación Profesional Stella Maris* (Stella Maris Career Guidance Centre) to conceal the true function of this facility. It could accommodate up to 33 inmates, who were isolated there for up to 3 months because of prostitution, drug abuse, theft, “participation in the hippie movement,” as well as “illicit relationships” or “abnormal behavior.” The windows of the Observation and Classification Centre were fitted with mental seals to prevent the inmates from escaping (19). Although the isolation was almost exclusively due to social misconduct on the part of the individuals concerned and had no medical causes, the young women nevertheless had to undergo extensive medical and psychological examinations upon arrival. The medical staff included a general practitioner, a gynecologist, a psychologist and a psychiatrist (20).

Initially, young women suspected of clandestine prostitution were referred to a venereal disease clinic in Barcelona for examinations and clinical tests. The directive of the Observation and Classification Centre viewed this procedure very critically because they feared that the patients could use their stay in the clinic to attempt to escape. For this reason, in October 1967, Dr. Josep Mercadal Peyrí (1903–1976) was commissioned to examine the young women for venereal diseases directly in the Observation and Classification Centre (21).

Mercadal Peyrí had married into a very influential Catalan family of venereologists and was himself head of dermatology both at the Sant Pere Claver Hospital in Barcelona and the Red Cross Hospital in L‘Hospitalet de Llobregat. As a devout Catholic, Mercadal Peyrí was well connected in religious scientific circles and served, among other things, as vice-president of the International Association of Catholic Doctors (22). There are no sources indicating what venereological examinations were performed at the Observation and Classification Centre by Mercadal Peyrí, who held his position until his retirement at the end of 1975 (23). The fact that blank referral forms existed for young women suspected of clandestine prostitution indicates that these examinations were requested frequently (24). Similar to Ribas Magri, Mercadal i Peyrí was also involved in the Provincial Board of the Patronato beyond his duties as a venerologist, serving as an official representative in one of its commissions (25).

### 3.4 The cooperation of the Patronato with the *Hospital de la Magdalena*

**Figure 4:**
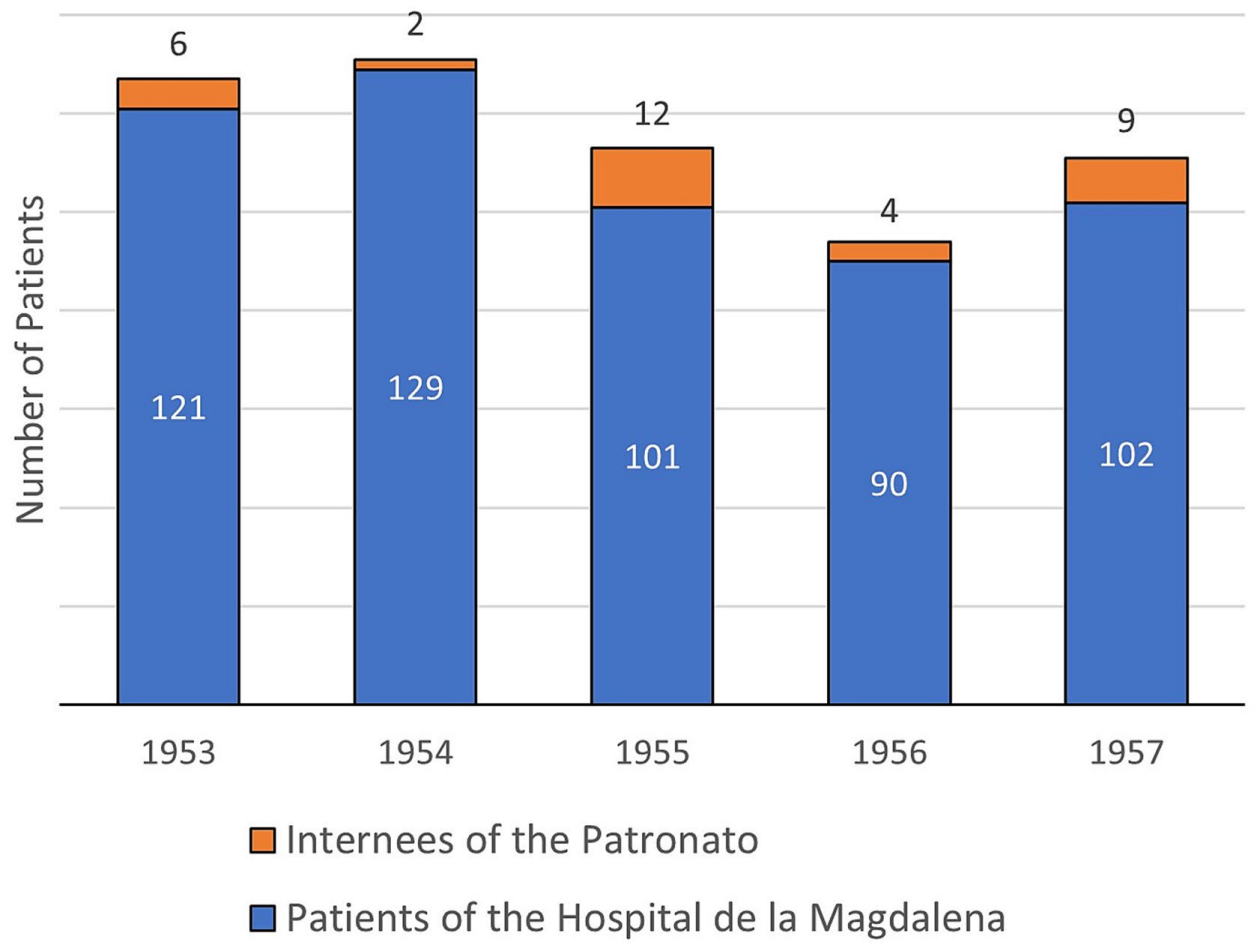
Proportion of young women under the guardianship of the Patronato among patients at the *Hospital de la Magdalena* between 1953 and 1957 (43–49).

**Figure 5:**
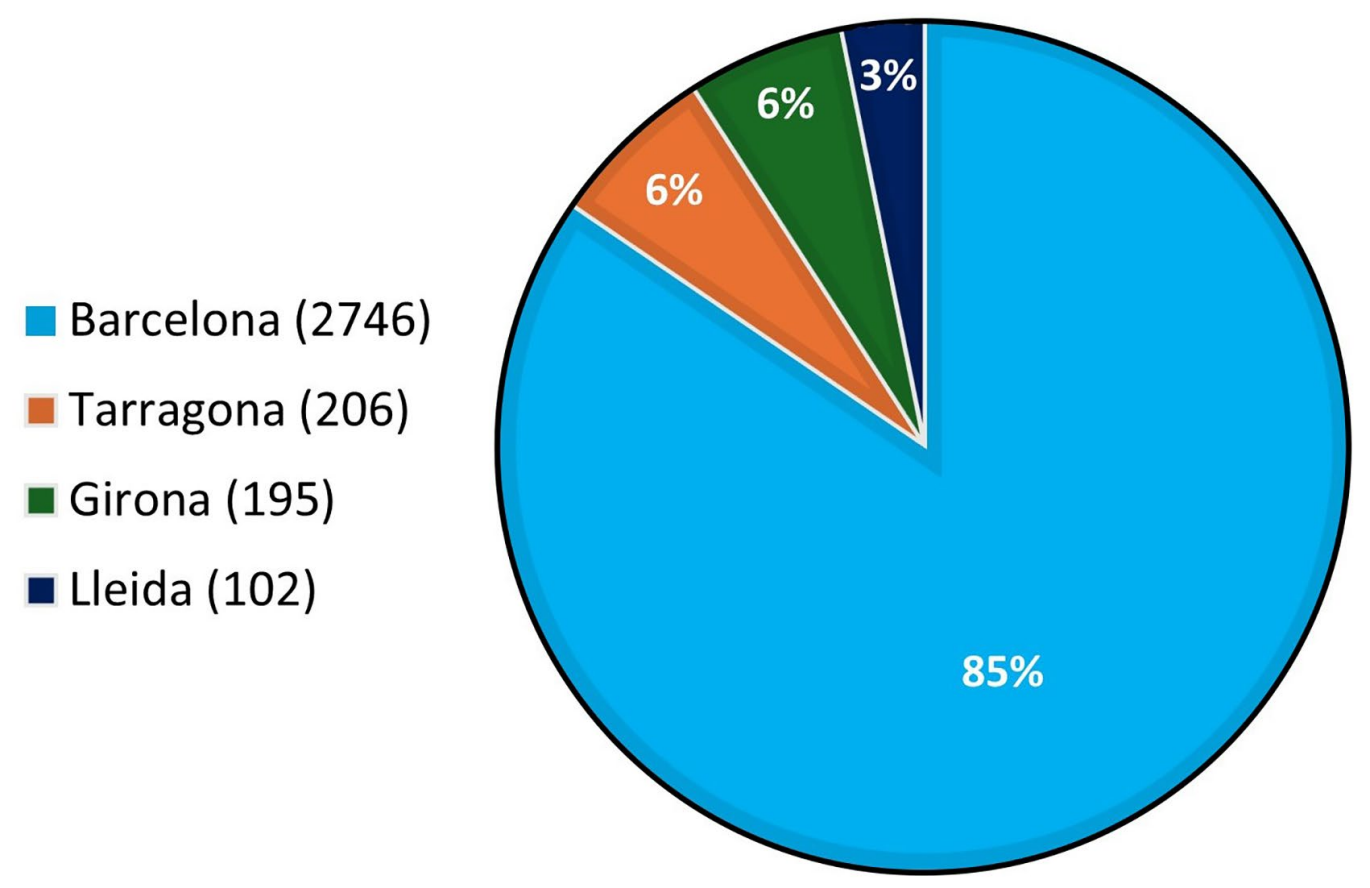
Admissions of young women to Patronato facilities in Catalonia up to 1965.

The Patronato not only commissioned doctors to carry out venereological examinations and treatments in its facilities, but also cooperated with medical institutions. Its most important partner in the field of venereology in Barcelona was the *Hospital de la Magdalena* (Figure 4). As Figure 5 illustrates, between 1953 and 1957 young women in the care of the Patronato were regularly admitted as patients to the *Hospital de la Magdalena*. The fluctuation in the numbers, particularly between 1954 and 1956, is difficult to interpret. It is noteworthy, however, that the proportion of Internees of the Patronato in 1955 and 1957 was around 10%, meaning they constituted a significant proportion of the total number of patients at the *Hospital de la Magdalena*.

When the *Hospital de la Magdalena* had been established in November 1923, it was the first hospital in Spain to treat exclusively female prostitutes hospitalized with venereal diseases (26). Women who were excused of prostitution were admitted by the police without legal basis if they were suspected of having a venereal disease (27, 28). A predecessor organization of the Patronato had already been cooperating with the *Hospital de la Magdalena* in the early 1930s (29–31). In the last months before the beginning of the Spanish Civil War, the director of the Catalan Association to Fight Venereal Diseases at that time, Antoní Peyrí Rocamora (1889–1973), the future uncle-in-law of the aforementioned Mercadal Peyri, made considerable efforts to provide moral and cultural education for prostitutes hospitalized in the *Hospital de la Magdalena* in addition to medical treatment (32).

Only one year after the founding of the provincial board of the Patronato in Barcelona, its steering committee already commissioned an informant to compile a dossier on the *Hospital de la Magdalena* to explore the basis for cooperation. The report concluded that due to the medical staff’s lack of moral and religious formation, it was currently almost impossible to carry out the education of the patients according to the ideology of the Patronato (51). In the following years, the Patronato managed to increase its influence over the hospital significantly. Its steering committee insisted that there should be a separate area for the other patients, who were considered to be incorrigible prostitutes that could exert a bad influence on the young women under the guardianship of the Patronato (33). Whether this request was granted cannot be answered on the basis of the available sources. In 1959, the *Hospital de la Magdalena* was closed on the grounds that improvements in methods meant that long-term hospital treatment for venereal diseases was no longer necessary (34).

### 3.5 Medical care in the other provincial boards of the Patronato in Catalonia

There were also provincial boards of the Patonato in the other three Catalan provinces. No contemporary documents regarding the provincial board of the Patronato in Tarragona exist. The very few surviving sources on the Patronato in Girona are also insufficient to reconstruct how the institutions there functioned and how the internees were medically cared for. Thus, based on the available material, only the provincial board of the Patronato in Lleida can be examined in similar detail as that in Barcelona. Based on the number of admissions to the facilities of the provincial boards of the Patronato up to 1965, it can be assumed that conditions in Girona and Tarragona were much more similar to those in Lleida than in Barcelona. In Barcelona, significantly more young women were referred to the Patronato than in all other Catalan provincial boards combined. These numbers suggest that the Patronato’s facilities, and consequently its medical services, were of far greater importance in large cities than in the more rural provinces (Figure 5).

The provincial board of the Patronato in Lleida was founded in October 1942. Non only the number of internments but also the medical staff was considerably smaller than in Barcelona and included only one doctor and a nurse. The doctor’s main task was to regularly examine the 30 prostitutes registered in Lleida’s three brothels for venereal diseases. There are no sources detailing how these examinations were conducted. As Lleida did not have its own Observation and Classification Centre, young women accused of prostitution or of another illegitimate relation with a man were sent by the provincial board of the Patronato in Lleida either to Madrid or to Barcelona for their initial examination (35). Like all other institutions of the Patronato in Catalonia, the provincial board in Lleida effectively only ceased to exist at the end of 1982, when it was placed under the authority of the Catalan regional government (36).

## 4 Discussion

In accordance with the directives of the national board in Madrid, medical services were established in all of the Patronato’s provincial boards in Catalonia. In Lleida, and presumably also in Tarragona and Girona, these services were limited to regular examinations of prostitutes for venereal diseases. In Barcelona, however, venereological examinations were carried out both at the headquarters of the provincial board and later at the Observation and Classification Centre.

The results of this study cast doubt on the medical indication of these examinations. Instead, the reason for the examinations was rather that the young women had not behaved in accordance with the traditional Catholic gender roles prescribed by the Franco dictatorship and were therefore suspected of prostitution and spreading sexually transmitted diseases. The venereologists at the service of the Patronato came from highly respected Catalan medical families and were involved in the provincial board of the Patronato far beyond their work as doctors. Due to the lack of sources, it is difficult to provide evidence that their political and social views influenced their clinical decisions. However, the documents analyzed indicate that these doctors were specifically selected by the Patronato as venereologists because of their close ties to the Catholic Church. At the *Hospital de la Magdalena*, where until 1959 young women were sent under the care of the Patronato if they had been diagnosed with a sexually transmitted disease, the focus was not solely on curing the patients. Instead, the Patronato attempted to exert its influence so that those hospitalized there received not only medical treatment but also moral and religious education.

*Limitations*: Medical records that might have provided insight into specific venereological practices were either unavailable or inaccessible due to legal restrictions. As the stigma surrounding those affected still lingers to this day, very few of them are willing to talk about their experiences. This made it difficult to gain insights into the patients’ perspective and to provide significant evidence that the venereological examinations performed differed from the contemporary standard medical protocols or that diagnoses were falsified for punitive reasons.

Since early modern times, people suffering from venereal diseases have been stigmatized and excluded from society. Coercive measures to combat sexually transmitted diseases were directed primarily against female sex workers and other marginalized social groups. As recent studies have shown, even in the second half of the 20th century, the fight against venereal diseases served as a pretext for social exclusion. In the German Democratic Republic and other socialist dictatorships, young women were isolated in closed venereological wards without any medical indication. There they were forcibly treated, even though most of them did not have a sexually transmitted disease at all. Instead, in most cases, the reason for their admission to these institutions was that they did not want to conform to the socialist image of women (37–39).

In research, the term “politicization of medicine” has become established to describe this form of misuse of medicine. It refers to a social process in which ideological views increasingly permeate medical practice and institutional frameworks. Medical examinations and treatments thereby lose their primarily therapeutic character and become linked to political objectives. This can occur both through direct state intervention and through the influence of ideological interpretative frameworks on medical decision-making processes. Such a development not only affects the autonomy of medical practice but can also have a lasting impact on the organization of healthcare and trust in medical institutions.

The case examined in this paper differs from existing research primarily in that Francoist Spain was not a socialist dictatorship, but rather a regime with fascist traits that propagated a traditional, Catholic image of women. Furthermore, the institutions examined were not closed venereological wards and thus medical facilities, but rather institutions run by the Catholic organization *Patronato de Proteccion a la Mujer*.

Although it is not possible to prove the existence of “politicized medicine,” the circumstances in which the venereological examinations and treatments were carried out strongly suggest that mechanisms of social control were at play. With regard to the venereological examinations carried out at the headquarters of the Patronato’s provincial board in Barcelona, it has been shown that these took place against the will of the underage women and their legal guardians. The examinations were not prompted by medical reasons, but by the fact that the young women were suspected of engaging in socially unacceptable contact with men. Nor was there any medically justified suspicion of a sexually transmitted disease in the case of the young women who were admitted to the Observation and Classification Centre and then referred to the venereologist employed by the Patronato. Instead, they were accused of secretly engaging in prostitution.

Despite its limitations, the findings of this study implicate a critical intersection of public health, medical ethics, and social control during the Franco dictatorship. In an increasingly radicalized political climate and public discourse, it is essential in public health ethics to highlight the dangers of doctors being guided by political ideologies rather than medical facts. This applies in particular to the treatment of vulnerable populations, including children and adolescents, who are unable to express their own will, or can do so only inadequately. They are particularly at risk of becoming victims of social control mechanisms and politicized medicine. Given that sexually transmitted infections have been on the rise again worldwide in recent years, it is also important to use historical examples to illustrate that patients suffering from such diseases are constantly at risk of stigmatization due to their specific modes of transmission.

## 5 Conclusion

This study strongly suggests that both the Patronato’s institutions examined in Catalonia and the medical institutions that collaborated with the Patronato carried out venereological examinations which were less medically indicated than they were part of social control mechanisms. The venereologists at the service of the Patronato not only performed their medical duties, but were also actively involved in the leadership of this institution whose traditional Catholic view of gender they shared. Due to the limited source material available, this study was unable to conclusively determine whether, in the case of Franco’s dictatorship, one can speak of “politicized medicine” in a similar way to the German Democratic Republic and other socialist regimes. To this end, it is necessary to analyze further sources from other regions of Spain regarding the Patronato’s collaboration with medical institutions. It would also be desirable to conduct interviews with contemporary witnesses to validate the victimization process from the perspective of those treated.

## Data Availability

The data analyzed in this study is subject to the following licenses/restrictions: the datasets presented in this article are not readily available because as the archival documents are the source of this article, we are not authorized to provide copies to third parties. However, these documents can be accessed by researchers if they contact the archives directly. Following the rules of good scientific practice, we have indicated the signatures of the archival documents, ensuring the transparency of our research. Requests to access these datasets should be directed to Florian Grafl, Florian-1.grafl@uni-ulm.de.
